# Accumulation of Inflammatory Mediators in the Normal Pericardial Fluid

**DOI:** 10.3390/ijms25010157

**Published:** 2023-12-21

**Authors:** Mohammad M. El-Diasty, Javier Rodríguez, Luis Pérez, Sonia Eiras, Angel L. Fernández

**Affiliations:** 1 Cardiac Surgery Department, Harrington Heart and Vascular Institute, University Hospitals Cleveland Medical Center, Cleveland, OH 44106, USA; mohammadmeldiasty@gmail.com; 2 Division of Clinical Biochemistry, University Hospital, 15706 Santiago de Compostela, Spain; javierrodriguez8@gmail.com (J.R.); luisfperez2@gmail.com (L.P.); 3 Laboratory of Cardiovascular Research, University Hospital, 15706 Santiago de Compostela, Spain; soniaeiraspenas@gmail.com; 4 Division of Cardiac Surgery, University Hospital, Department of Surgery, University of Santiago de Compostela, 15706 Santiago de Compostela, Spain

**Keywords:** cytokines, inflammation, interleukins, interleukin 1α (IL-1α), interleukin 1β (IL-1β), interleukin 2 (IL-2) interleukin 4 (IL-4), interleukin 6 (IL-6), interleukin 8 (IL8), interleukin 10 (IL10), tumor necrosis factor-α (TNF-α), interferon-γ, pericardium, pericardial fluid, growth factors, adhesion molecules

## Abstract

There is paucity of studies that focus on the composition of pericardial fluid under resting conditions. The purpose of this study is to determine the levels of inflammatory mediators in pericardial fluid and their correlation with plasma levels in patients undergoing elective cardiac surgery. We conducted a prospective cohort study on candidates for elective aortic valve replacement surgery. Pericardial fluid and peripheral venous blood samples were collected after opening the pericardium. Levels of interleukin 1α (IL-1α); interleukin 1β (IL-1β); interleukin 2 (IL-2) interleukin 4 (IL-4); interleukin 6 (IL-6); interleukin 8 (IL8); interleukin 10 (IL10); tumor necrosis factor-α (TNF-α), interferon-γ (IFN-γ), vascular endothelial growth factor (VEGF), monocyte chemotactic protein-1 (MCP-1) epidermal growth factor (EGF), soluble E-selectin, L-selectin, P-selectin, intercellular adhesion molecule-1 (ICAM-1), and vascular cell adhesion molecule-1 (VCAM-1) were determined in both pericardial fluid and serum samples. A total of 45 patients with a mean age of 74 years were included of which 66% were males. Serum levels of all study mediators were within normal limits. Serum and pericardial levels of IL-1 α, IL-1 β, IL-2, IL-4, and IL-10 were similar. Levels of VEGF, EGF, VCAM-2, ICAM 1, E-selectin, P-selectin, and L-selectin were significantly lower in pericardial fluid than in serum. However, levels of IL-6, IL-8, TNF-α, IFN-γ, MCP-1, and MCP-1 were significantly higher in the pericardial fluid than in serum. Under normal conditions, the pattern of distribution of different inflammatory mediators in the pericardial fluid does not reflect serum levels. This may either reflect the condition of the underlying myocardium and epicardial fat or the activity of the mesothelial and mononuclear cells present in pericardial fluid.

## 1. Introduction

Cardiac interstitial fluid diffuses into the pericardial cavity through the epicardium and is the main component of the physiological pericardial fluid. The epicardium acts as a semi-permeable membrane allowing the passage of substances with a molecular weight of less than 40 kDa. Physiological pericardial fluid is considered an ultrafiltrate of serum and contains an overall protein concentration lower than in serum [1,2,3,4]. The myocardium, the stromal cells, and the epicardial fat are known to synthesize peptides including inflammatory mediators which may exert both autocrine and paracrine functions [5,6]. After their production, these proteins can be found at the cardiac interstitial microenvironment where they can be redistributed to the systemic circulation via the cardiac capillaries and venous drainage network. Also, the visceral epicardium allows the diffusion of these proteins from the interstitial fluid directly into the pericardial space [7,8,9,10,11]. In addition, the mesothelial cells are known to secrete different substances directly into to the pericardial cavity [12].

The objective of this study is to determine the levels of a variety of inflammatory mediators in the pericardial fluid of patients without pericarditis or pericardial effusion and their correlation with serum levels.

## 2. Results

Forty-five consecutive patients who fulfilled the inclusion criteria were included in this study. The mean age was 74.5 ± 6.1 years. Table 1 summarizes the clinical data of the included patients.

Table 2 summarizes the values of interleukins and growth factors in both pericardial fluid and serum.

Table 3 summarizes the values of soluble adhesion molecules in both pericardial fluid and serum.

Serum levels of all study mediators were within normal limits.

There was no difference between the pericardial fluid and serum levels of interleukins IL-1α, IL-1β, IL-2, IL-4, and IL-10.

Significantly lower levels of VEGF, EGF, VCAM-2, ICAM-1, E-selectin, P-selectin, and L-selectin were found in pericardial fluid.

Significantly higher levels of interleukins IL-6, IL-8, TNF-α, IFN-γ, and MCP-1 were found in the pericardial fluid.

Our results demonstrate that there is an accumulation of some inflammatory mediators in the pericardial fluid in the absence of pericardial effusion or pericarditis independently on their molecular weight. These inflammatory mediators can be synthesized in the cardiopericardial compartment.

Regarding the cellular component in the pericardial fluid, the mean value of cell count was 2.4 ± 0.8/µL. The immunocytochemical analysis demonstrated that the dominant cell phenotype was histiocyte/monocyte (identified by immunostaining against CD68) and mesothelial cell (identified by immunostaining against cytokeratine).

## 3. Discussion

The biochemical study of the pericardial fluid obtained during a diagnostic or therapeutic pericardiocentesis in patients with pericardial effusion is a common procedure. However, few studies evaluate the biochemical composition of pericardial fluid in the absence of pericarditis or effusion. The main reason behind the scarcity of studies analyzing physiological pericardial fluid is the difficulty of collecting fluid samples. Therefore, all studies of physiological pericardial fluid were conducted in patients undergoing cardiac surgery [1,2,13].

We observed that the serum levels of interleukins, growth factors, and adhesion molecules were within the normal range. However, when comparing serum and pericardial levels, we found that there was a significant discrepancy in cases of some of these mediators.

While there was not any significant difference between serum and pericardial fluid levels of interleukins IL-1α, IL-1β, IL-2, IL-4, and IL-10, serum levels were higher than in pericardial fluid in cases of VEGF, EGF, and all soluble adhesion molecules. In contrast, pericardial fluid levels were higher in cases of interleukins IL-6, IL-8, INF-γ, TNF-α, and MCP-1.

There are previous studies that measured levels of some inflammatory mediators in the pericardial fluid obtained during cardiac surgery. While pericardial levels of IL-6, IL-8, TNF-α, and MCP-1 were reportedly higher in pericardial fluid than in serum, there was no significant difference in the levels of IL-1β [8,14,15,16,17,18].

Regarding VEGF levels, previous studies found higher levels in serum than in pericardial fluid [19,20,21]; however, this difference was not demonstrated by other groups [22]. Regarding INF-α, to the best of our knowledge, there are no previous reports of its levels in patients without pericardial effusion. However, Liu and colleagues reported higher levels of INF-α in pericardial effusions in patients with pericarditis [23]. In relation to adhesion molecules, only P-selectin levels were previously studied with similar findings to our present study (higher in serum than in pericardial fluid) [18]. From one side, the molecular weight and structure of the proteins determine their ability to diffuse from the cardiac interstitial space into the pericardial sac via the visceral epicardial layer [24]. In this sense, it was found that proteins with small molecular weights, typically less than 40 kDa, can easily diffuse into the pericardial space. This may explain the similarity between pericardial fluid and serum levels of certain small proteins such as IL-1α, IL-1β, IL-2, IL-4, and IL-10. However, this does not explain why the levels of other small proteins such as IL-6, IL-8, IL-10, INF-α, and MCP-1 are higher in pericardial fluid.

From the other side, it is known that epicardial adipose tissue as well as cardiomyocytes and stromal cells can express inflammatory mediators such as IL-6 and MCP-1 [25,26]. Therefore, the local synthesis of inflammatory mediators at the cardiac tissue level and their continuous leak from the interstitial space into the pericardial cavity may explain the high levels of these proteins in the pericardial fluid.

Also, it is possible that these mediators may be directly released by the pericardial fluid mononuclear cells or by the mesothelium lining of the pericardial cavity. In fact, in our study, the cellular population of the pericardial fluid was characterized by the abundance of macrophages and mesothelial cells. This hypothesis is supported by the finding that epicardial mesothelial cells under normal conditions are able to synthesize IL-6 [12] while the T lymphocytes present in the pericardial fluid are capable of synthesizing IL-6, IL-10, and INF-γ [18].

Conversely, the large molecular weights of VCAM-1, ICAM-1, E-selectin, P-selectin, L-selectin, and VEGF impede their diffusion through the epicardium and therefore their pericardial levels are lower than in serum.

However, the relation between membrane diffusion and molecular weight does not explain why EGF, a small protein of 6.8 kDa that would be expected to diffuse easily into the pericardial fluid, had a lower pericardial level than serum. The same happens regarding myoglobin (15 kDa) as lower pericardial concentrations were reported in previous studies [27]. EGF and myoglobin should be expected to migrate into the pericardial cavity due to their small molecular size. We hypothesize that the proportions of EGF and myoglobin released from the cardiopericardial compartment into the pericardial cavity are lower than that released from the rest of the body tissues and fluids (saliva, urine, milk, and peripheral skeletal muscle) to the peripheral circulation. This may explain why the overall plasma concentration of these proteins is higher than in pericardial fluid.

The main limitation of this study is that pericardial fluid was obtained from patients undergoing cardiac surgery. For obvious ethical reasons, physiological pericardial fluid cannot be obtained from completely healthy individuals. Therefore, to ascertain that the pericardial fluid’s composition was as close to “normal” as possible, we only enrolled stable patients undergoing elective cardiac surgery who had no ongoing myocardial ischemia or heart failure or any other parameters indicative of pericardial disease.

## 4. Materials and Methods

This is a prospective cohort study that includes patients with aortic stenosis who are candidates for elective aortic valve replacement surgery. Exclusion criteria were evidence of pericardial disease, previous cardiac surgery, presence of ischemic heart disease, diabetes mellitus, end-stage kidney dysfunction on dialysis, history of inflammatory or autoimmune diseases, atrial fibrillation, and recent consumption of anti-inflammatory medications. The study protocol conformed to the ethical guidelines of the 1975 Declaration of Helsinki as reflected in a priori approval by the Institutional Ethics Committee. A written informed consent was obtained according to the protocol approved by the Institutional Ethics Committee.

All surgical interventions were performed via median sternotomy. Before giving heparin, the pericardium was incised vertically, and a 5–10 mL sample of pericardial fluid was gently withdrawn using a 10 mL syringe and a 14 G catheter. Special precaution was taken to avoid sample contamination with blood. Simultaneous peripheral venous sample was also extracted. Both samples were transferred to sterile tubes that were kept on ice while being transferred immediately to the laboratory.

After separating the cellular components by centrifugation at 3500 rpm for 10 min at 4 °C, the supernatant fluid was isolated and stored at −30 °C. Interleukins, growth factors, and adhesion molecules were simultaneously measured in serum and pericardial fluid using the Evidence^®^ biochip array technology (Randox Laboratories Ltd., Crumlin, Antrim, UK). This biochip array technology uses the sandwich chemiluminescent immunoassay to detect levels of multiple analytes at the same time from a single sample. Two different multi-analyte panels were used in this study: The Evidence^®^ Cytokine and Growth Factors array was used for simultaneous quantitative detection of interleukins IL-1α, IL-1β, IL-2, IL-4, IL-6, IL-8, IL-10, tumor necrosis factor-α (TNF-α), interferon-γ (IFN-γ), vascular endothelial growth factor (VEGF), monocyte chemotactic protein-1 (MCP-1), and epidermal growth factor (EGF). The Evidence^®^ Adhesion Molecules array was used for the quantitative detection of soluble E-selectin, L-selectin, P-selectin, intercellular adhesion molecule-1 (ICAM-1), and vascular cell adhesion molecule-1 (VCAM-1).

Samples management and immunoassays were conducted on the automated biochip array analyzer according to the manufacturer’s instructions as previously described [28,29,30].

A minimum of 500 µL of sample is required for standard sample cups of Cytokine and Growth Factors array. Reagent composition includes: Cytokine Assay Diluent: 20 mM Tris-buffered saline pH 7.2 containing a protein matrix, detergent, and preservatives; Cytokine Conjugate: 20 mM Tris-buffered saline pH 7.5 containing a protein matrix, detergent, preservatives, and assay specific antibodies labeled with horseradish peroxidise (HRP), and Cytokine Biochips: Solid-phase substrate containing an immobilized antibody in discrete test regions. Instrument calibration is performed using the Randox Cytokine calibrators. Calibration is performed upon initial setup of the system. Intermittent calibration of the system is also performed to ensure accurate and reliable results. A multi-point calibration is conducted with the change in reagent lot, or as indicated by quality control procedures. Results are processed automatically using dedicated software 2.0.0.

Samples are diluted 1 in 10 using working strength wash buffer (50 µL of sample added to 450 µL of wash buffer and mixed thoroughly) for the determination of adhesion molecules. Minimal volume required is 25 µL of serum. Reagent array composition includes: Adhesion Molecules Assay Diluent: 19 mM Tris-buffered saline, pH 7.2 containing a protein matrix, surfactant, and preservatives; Adhesion Molecules Conjugate: 19 mM Tris-based buffer, pH 7.5 containing a protein matrix, surfactant, preservatives and assay specific antibodies labeled with horseradish peroxidase (HRP), and Adhesion Molecules Biochips: Solid-phase substrate containing discrete test regions of an immobilized antibody. A nine-point calibration is performed using the Adhesion Molecules Evidence^®^ calibrators. Calibration is also performed upon initial setup of the system. Intermittent calibration of the system is necessary to ensure accurate and reliable results. A multi-point calibration is performed with the change in reagent lot, or as indicated by quality control procedures. Results are processed automatically using dedicated software 2.0.0. However, patient sample results must be manually multiplied by 10 to account for sample dilution.

A sample aliquot of pericardial fluid was used for cellular count (DVIA 120^®^ Hematology System (Siemens Healthcare Diagnostics, Deerfield, IL, USA) and cytological study using two types of stain: Hemacolor^®^ (Merck KGaA, Darmastat, Germany) and Papanicolau using the automatic stain system Hema-Tek^®^ (Bayer Healthcare Ag, Leverkusen, Germany). The immunochemical study was realized through the avidine–biotine complex (ABC) technique. The automatic immunostainer Dako Cytomation Autostainer Techmake 500 Plus^®^ (Dako, Glostrup, Denmark) was used. Immunostaining was realized against CD68 (Dako, Glostrup, Denmark) dilution 1:100 and against cytokeratin AE1.AE3 (Dako, Glostrup, Denmark) dilution 1:50.

### Statistical Analysis

Categorical variables were expressed as numbers and percentages. Continuous variables were presented as means ± standard deviation (SD) and were compared by using 2-tailed Student’s *t* test or Mann–Whitney test. A *p* value of <0.05 was considered statistically significant. The software SigmaStat 3.1 was used (Systat Software, Inc., San Jose, CA, USA).

## 5. Conclusions

Under normal conditions, there is an accumulation of IL-6, IL-8, IL-10, INF-γ, and MCP-1 in the pericardial fluid. It, however, remains unclear if this enhanced accumulation reflects the physiological condition of the cardiac interstitial tissue or if it results from the local synthesis of mediators by mesothelial and mononuclear cells present in the pericardial fluid.

## Figures and Tables

**Table 1:** Demographic and preoperative characteristics of the patients.

Age (years)	74.5 ± 6.1
Sex female/male (n)	15/30
BMI (kg/m^2^)	28.7 ± 3.1
Hypertension (%)	42.2
LVEF (%)	54 ± 7
Total protein (g/dL)	6.4 ± 0.6
Albumin (g/dL)	4.2 ± 0.3
Creatinine (mg/dL)	1 ± 0.2
Hemoglobin (g/dL)	13.5 ± 1.1

BMI: Body Mass Index (weight kg/height m^2^); LVEF: Left ventricular ejection fraction.

**Table 2:** Serum and pericardial fluid concentrations of interleukins and growth factors.

	Serum	Pericardial Fluid	*p* Value
IL-1α (pg/mL)	0.12 ± 0.21	0.140 ± 0.29	0.632
IL-1β (pg/mL)	0.29 ± 0.15	0.48 ± 0.6	0.731
IL-2 (pg/mL)	2.71 ± 3.14	8.23 ± 11.45	0.534
IL-4 (pg/mL)	6.78 ± 2.55	5.85 ± 4.82	0.281
IL-6 (pg/mL)	11.49 ± 6.08	183.81 ± 94.49	<0.01
IL-8 (pg/mL)	7.82 ± 5.69	28.12 ± 20.92	<0.01
IL-10 (pg/mL)	0.25 ± 0.24	0.52 ± 0.41	0.396
TNF-α (pg/mL)	3.18 ± 1.06	4.271 ± 0.28	<0.05
IFN-γ (pg/mL)	0.83 ± 0.65	5.34 ± 5.12	<0.01
VEGF (pg/mL)	163.35 ± 129.21	22.8 ± 16.36	<0.01
MCP-1 (pg/mL)	301.6 ± 58.32	1206.98 ± 285.49	<0.01
EGF (pg/mL)	81.57 ± 40.78	2.97 ± 1.67	<0.001

IL-1α: interleukin 1α; IL-1β: interleukin 1β; IL-2: interleukin 2; IL-4: interleukin 4; IL-6: interleukin 6; IL-8: interleukin 8, IL-10: interleukin 10, TNF-α: tumor necrosis factor-α; IFN-γ: interferon-γ; VEGF: vascular endothelial growth factor; MCP-1: monocyte chemotactic protein-1; EGF: epidermal growth factor (EGF).

**Table 3:** Serum and pericardial fluid concentration of soluble adhesion molecules.

	Serum	Pericardial Fluid	*p* Value
VCAM-1 (ng/mL)	1245.5 ± 301.2	218.3 ± 148.3	<0.01
ICAM-1 (ng/mL)	568.4 ± 137.7	301.7 ± 75.1	<0.05
E-selectin (ng/mL)	17.44 ± 3.82	3.8 ± 1.75	<0.01
P-selectin (ng/mL)	272.26 ± 51.3	19.35 ± 2.6	<0.01
L-selectin (ng/mL)	1486.92 ± 208.5	695.1 ± 177.3	<0.01

ICAM-1: intercellular adhesion molecule-1; VCAM-1: vascular cell adhesion molecule-1.

## Data Availability

The data presented in this study are available on request from the corresponding author. The data are not publicly available due to ethical restrictions.
